# Hermansky-Pudlak Syndrome; a Case Report

**Published:** 2010-10

**Authors:** Abbas Bagheri, Asieh Abdollahi

**Affiliations:** Ophthalmic Research Center, Labbafinejad Medical Center, Shahid Beheshti University of Medical Sciences, Tehran, Iran

**Keywords:** Albinism, Bruising, Hermanski-Pudlak Syndrome, Strabismus

## Abstract

**Purpose:**

To report a case of Hermansky-Pudlak syndrome.

**Case Report:**

A seven-year-old boy presented with marked generalized hypopigmentation, ocular exodeviation and nystagmus. He had history of easy bruising. Examination revealed green irides with marked transillumination, hypopigmented fundi and foveal hypoplasia. Further investigations disclosed platelet storage defect with adenosine diphosphate deficiency and abnormal aggregation compatible with Hermansky-Pudlak syndrome. The patient underwent strabismus surgery taking necessary precautions such as reserving platelet concentrates in case of a hemorrhagic event.

**Conclusion:**

Patients with albinism should be evaluated for Hermansky-Pudlak syndrome especially before surgery to prevent life-threatening complications.

## INTRODUCTION

Hermansky-Pudlak syndrome (HPS) is an autosomal recessive disorder characterized by oculocutaneous albinism, platelet storage-pool deficiency and lysosomal accumulation of ceroid lipofuscin.^1^–^3^ The albinism results in iris transillumination, variable degrees of skin and hair hypopigmentation and congenital nystagmus.^4^ The storage-pool defect arises from the absence of platelet dense bodies which normally contain adenosine diphosphate (ADP), adenosine triphosphate (ATP), calcium and serotonin, and trigger the secondary aggregation response of platelets.^2^ Patients with HPS demonstrate easy bruising and prolonged bleeding after surgical procedures. The accumulation of ceroid lipofuscin, an amorphous lipid-protein complex, is associated with pulmonary fibrosis^5^,^6^ and granulomatous colitis.^7^ The bleeding diathesis is usually mild. However, death from hemorrhage has been reported.^8^ There is a high incidence of strabismus accompanying this syndrome, often requiring surgery^9^. Preoperative diagnosis of HPS helps minimize potential complications resulting from the bleeding diathesis during extraocular muscle surgery. Herein we report a case of HPS and its management.

## CASE REPORT

**Figure 1:**
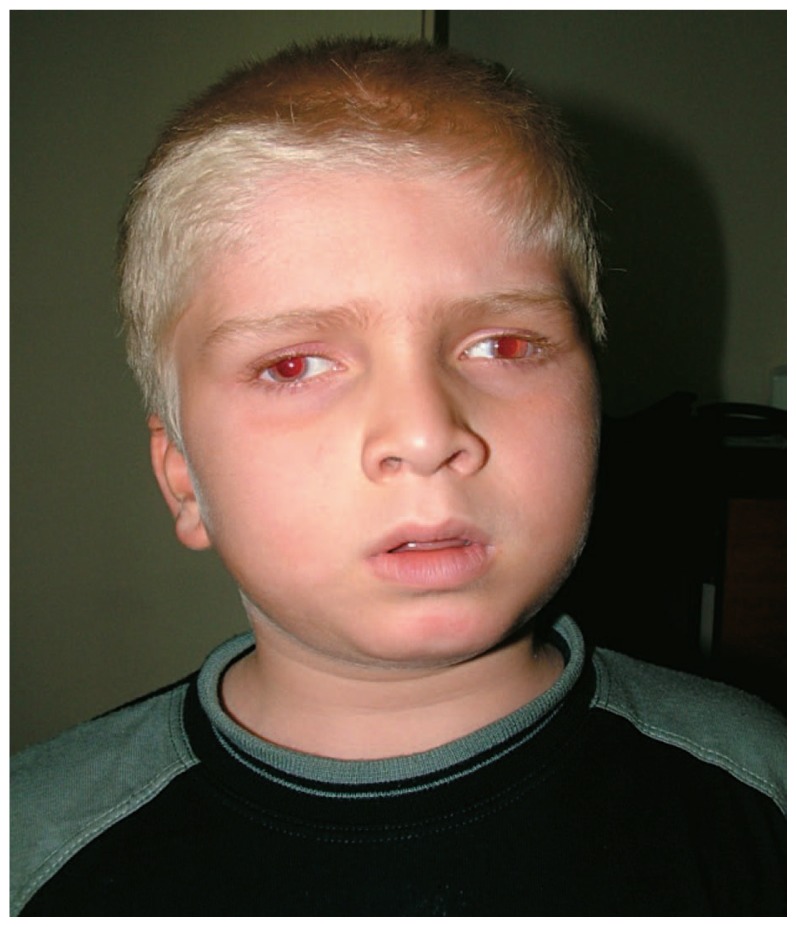
Patient at presentation, with oculocutaneous albinism and exotropia.

**Figure 2:**
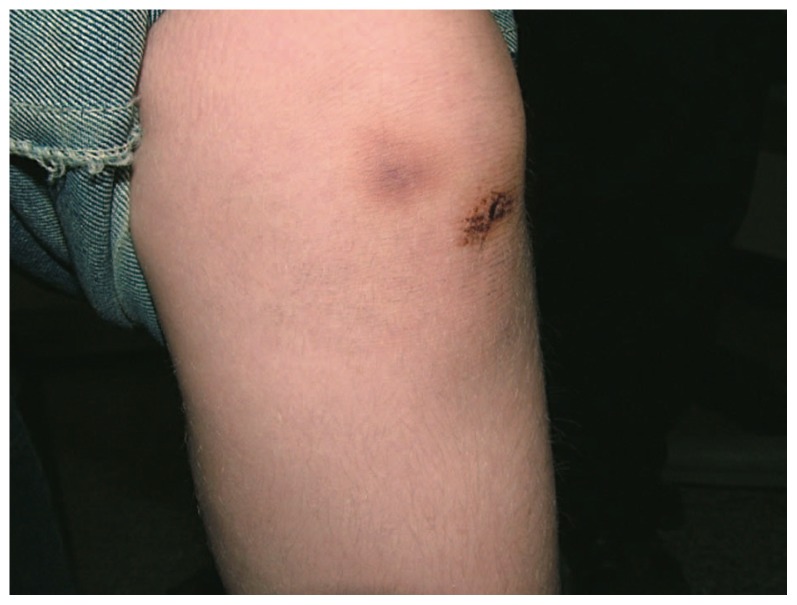
Bruising with minor trauma is seen over the left knee.

**Figure 3:**
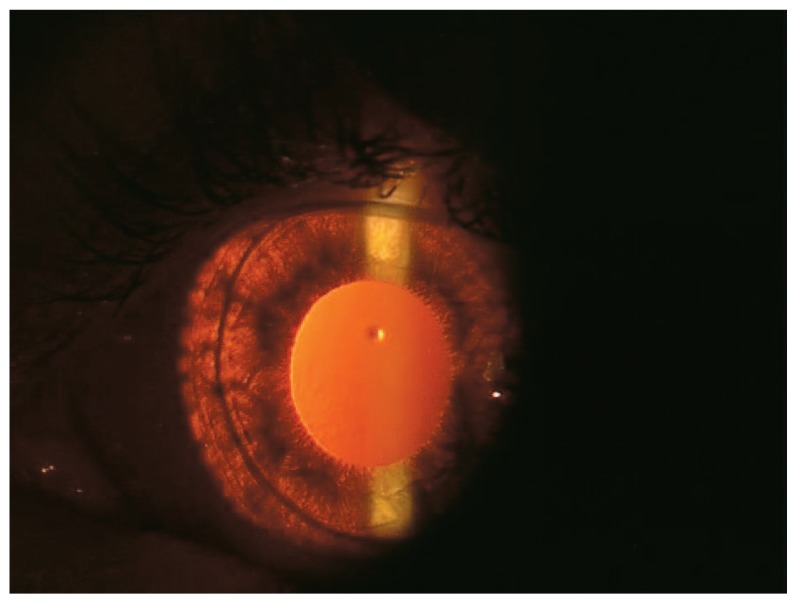
Transillumination of the iris.

A seven-year-old boy presented with marked generalized hypopigmentation, reduced visual acuity, photophobia, and nystagmus (Fig. 1). He had always bruised easily but superficially with minor trauma, without history of internal organ bleeding (Fig. 2). His parents were unrelated, and he had an unaffected brother and sister. Best corrected monocular visual acuity was 20/200 in both eyes. Due to presence of nystagmus, monocular vision was measured by fogging the fellow eye with +6 D overcorrection. Binocular visual acuity was 20/160 when he maintained fusion. Refraction was +10.00-4.00×10° in the right and +9.00-4.00×160° in the left eye. He had obvious horizontal jerk nystagmus and 50 prism diopters (PD) of intermittent exotropia. His green irides were markedly transilluminable (Fig. 3), both fundi were hypopigmented and foveal hypoplasia was evident. The patient was found to have a platelet storage-pool defect together with prolonged bleeding time and abnormal platelet aggregation studies. His bleeding time was prolonged, however prothrombin time (PT), partial thromboplastin time (PTT) and platelet counts were normal. There was severe platelet release defect with low ADP and increased ATP/ADP ratio. A classic method of platelet aggregometry was performed. In this method, a panel of platelet agonists (collagen, ADP, and ristocetin) at a range of concentrations, triggers platelet activation and is used to detect storage-pool and secretion defects. Von Willebrand factor (vWF) activity was 76%, vWF antigen was 68%, and factor VIII activity was 55% (all values within normal limits). Tyrosinase activity was not measured.

**Figure 4:**
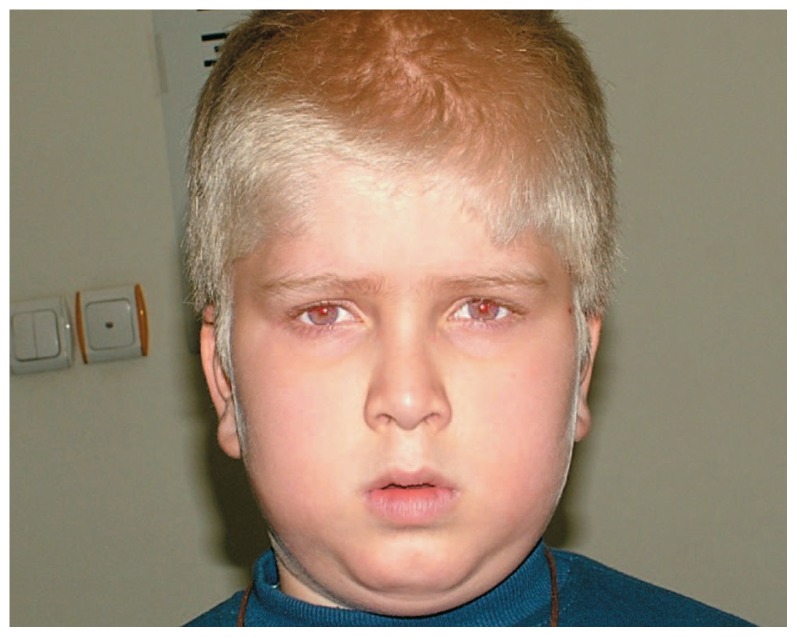
Improved exotropia after the operation.

The patient underwent strabismus surgery considering precautions such as reserving platelet concentrates in case of a hemorrhagic event. A 10 mm recession of both lateral recti was performed. Exotropia was reduced to 35 PD postoperatively. Three months later, a 6 mm resection of both medial rectus muscles was performed. After the second operation, the eyes became nearly orthophoric (Fig. 4). Visual acuity increased to 20/120 and nystagmus was decreased subjectively and objectively. Although intraoperative conjunctival bleeding was more than usual, platelet transfusion was not required. After 18 months, the eyes were nearly orthophoric and the patient was happy with the results.

## DISCUSSION

The degree of pigmentation of the skin, hair, iris and fundus varies significantly among patients with Hermansky-Pudlak syndrome. It has been suggested that ocular pigmentation increases with age^9^ and there is wide variation in the phenotypic expression of the condition.^10^

Nystagmus is always present in these patients.^4^,^9^,^11^ However, due to foveal hypoplasia, abnormal head posture is not present. Strabismus is a common finding in all forms of albinism. Esotropia is the most frequent deviation in this syndrome^9^–^11^, however our patient had exotropia.

The refractive profile of albino subjects, typified by high refractive errors with an overall bias toward hyperopia and high with-the-rule astigmatism, suggests impairment of normal physiologic emmetropization. However, there are discrepancies between studies in terms of associated refractive errors with both myopia^12^,^13^ and hyperopia^14^,^15^ being reported. In tyrosinase-positive forms of oculocutaneous albinism such as Hermansky-Pudlak syndrome, hyperopia is more common.^16^ Our patient had high hyperopia and with-the-rule astigmatism.

It has been shown that recession of all four horizontal rectus muscles can improve visual acuity and decrease nystagmus. By revising the surgical plan, strabismus can simultaneously be corrected.^17^,^18^ Although we had to resect both medial rectus muscles to correct residual exotropia, this procedure did not adversely affect visual function.

Bleeding is the leading cause of mortality in patients with HPS.^19^,^20^ Platelet function should be evaluated prior to surgery in all patients with oculocutaneous albinism to rule out HPS. A preoperative hematology consultation is advisable for patients with HPS. The bleeding diathesis in HPS is usually mild and has been reported to be controlled by prophylactic administration of desmopressin.^19^ Aspirin and indomethacin are contraindicated in patients with HPS, since they exacerbate the platelet abnormality.^20^ Platelet concentrates should be available in case severe bleeding occurs during or after surgery.
